# A Bayesian approach to investment in innovation projects with the presence of fake innovators

**DOI:** 10.1016/j.heliyon.2020.e05603

**Published:** 2020-11-25

**Authors:** Alim Gurtuev, Elena Derkach, Salima Makhosheva, Zaur Ivanov

**Affiliations:** Kabardino-Balkarian Scientific Center of Russian Academy of Sciences, Institute for Computer Science and Problems of Regional Management, 37a I.Armand st., Nalchik, Russia

**Keywords:** Decisions under uncertainty, Innovation, Funding, Bayesian games, Knowledge asymmetry, Economic development, Microeconomics, Infrastructure development, Risk management, Research and development, Economics, Information science

## Abstract

The paper proposes a game-theoretic model of interaction between investors and innovators, taking into account the existence of so-called “fake” innovators offering knowingly unprofitable projects. The model is a Bayesian non-cooperative, repetitive game with recalculated payments and partly unobservable *ex interim* player types. It allows quantifying the parameters of the strategy for all player types to find equilibrium solutions. The model describes rational modes for screening “fake” innovators based on adjusting players' probabilistic estimates.

## 1 Introduction

At present, a transformation of the industrial structure of the world economy is underway. Productions of intangible assets take an increased share in the gross domestic product (GDP), even the most traditional industries experience rapid technological changes, and startups of various types are rising everywhere. Increased mobility of factors of production induces relocation of industrial production into the “catching up growth” countries, and the knowledge and technology production remain in the OECD countries (Chen et al., 2010; Chemmanur and Simonyan, 2010).

This shift in world production structure adds to the interest of the mechanisms of investment in innovation projects, particularly in the theory of optimal mechanisms under uncertainty. It should be noted here that both parties are unable to produce an objective evaluation of the innovation project (Gurtuev et al., 2018). Moreover, current mechanisms seem to be quite ineffective, with less than 10% of innovation projects are funded, and a lot of them did not see any success in implementation. The possibility of building an optimal mechanism or even increasing the effectiveness of existing empirical mechanisms remains an open problem. It is crucial here that the sources of information incompleteness are not only the uncertainty of the future but also *a priori* biases by innovators of their projects, the asymmetry of knowledge, and much of noise in the evaluations of expertise coherence.

One of the critical issues in the investment in innovation projects is the detection of fraudulent (“fake”) projects. We define them as projects based on deliberately unrealizable concepts, which are aimed only at obtaining funding, and then, after a few years, either admit the impossibility of completing the project for objective reasons or create a product that does not have the declared characteristics. The following are some examples from recent history. *Theranos* is probably the best-known startup fraud, with a total of about $ 1.1 billion in funding. The company proposed new technology for blood analysis that uses much less blood, cheating investors for more than ten years (Hartmans and Leskin, 2020). Other projects include *Quintillion*, which received more than $ 270 million for the installation of a trans-Arctic submarine fiber optic cable (Peters, 2019), *Pixelon* that raised more than $30 million for an online video company that did not even have streaming technology, *Crescent Ridge Capital Partners* which was a Ponzi scheme with more than 50 defrauded investors and $7.5 million in damages, *Bouxtie* which raised more than $ 2.5 million for fake proposals of personalized gift cards, *WrkRiot*, *Asenqua Ventures*, and many other (CBInsights, 2019).

In this paper, we propose a game-theoretic model of interaction between the investor and the innovator in conditions when there are some fake innovators on the market. This is how this model differs from the common approach to evaluating innovation projects when it is assumed that uncertainty about the quality of the project is completely aleatoric (Nie et al., 2018). It should be noted that a similar approach, taking into account the possibility of deceptive behavior and distinguish it from pure uncertainty, is present in some recent works about bargaining (Kim, 2019). Unlike game models of the market with information asymmetry (Akerlof, 1970; Netzer and Scheuer, 2012; Pecorino and Van Boening, 2018; Lester et al., 2019), we assume multi-period bargaining game with the Bayesian adjustment of subjective assessments.

As a result of the model analysis, we get the conditions for the investor to make a decision on choosing an optimal strategy for the current game period. These conditions depend only on the parameters either directly observed or subjectively estimated by the investor.

The rest of the article is organized as follows: Section 2 provides a brief review of the literature, Sections 3 and 4 describe the basic approach and the essence of the study objective, Section 5 presents the main result in the form of a game-theoretic model and the results of model analysis, Section 6 contains conclusions and discussion of conclusions.

## 2 A brief review of related literature

As the state of the art shows, most works focused on the study of the individual decisions under risk or (mostly epistemic) uncertainty and modeling of particular markets (Arve and Martimort, 2016; Bergemann et al., 2018). Modern research on risk and uncertainty is mostly based on the utility theory approach and uncertainty being measured as an assessment of risk (Strausz, 2017; Doraszelski et al., 2018; Bergemann et al., 2018).

The violation of the efficiency of resource allocation in the conditions of information asymmetry has been proven in the literature many times (Akerlof, 1970; Spence, 1973; Netzer and Scheuer, 2012; Morris and Shin, 2012; Hackmann et al., 2015). Credit and insurance markets (Fama, 1980; Finkelstein and McGarry, 2006), secondary markets for goods with hidden characteristics (Greenwald et al., 1984; Garcia-Flores et al., 2000), and the labor market (Denzau and Munger, 1986; Cho and Kreps, 1987; Docquier and Marfouk, 2004) are usual examples. The main object of analysis there is the possible reaction of the particular market to asymmetric information, as well as the prerequisites for government intervention in the economy to correct market failures. Most of the studied problems with the information asymmetry can be divided into two classes in accordance with the temporal characteristics of information asymmetry – problems with unobservable (hidden) characteristics, where informational asymmetry is present before but vanishes after the trade, and unobservable actions when informational asymmetry is still in power after the trade. It should be noted that from the investor's point of view, the decision to invest (or not) in a particular innovation project is mainly a problem with unobservable characteristics and can be formally described as such a problem.

In a market with unobservable characteristics, research usually focuses on the possibility of partial or complete market collapse (adverse selection models). There are a significant number of papers studying the equilibrium in the labor market under conditions under unobservable types of workers, including the possibility of multiple equilibria and the problem of coordination. These studies led to the creation of the information theory of discrimination in the labor market (Denzau and Munger, 1986; Cho and Kreps, 1987; Hart and Moore, 1988). A significant number of papers are also devoted to discussing the problem of unobservable characteristics in credit and insurance markets and the possibility of disequilibrium and rationing as a market reaction to the information asymmetry (Fama, 1980; Guiso and Parigi, 1999; Garcia-Flores et al., 2000; Finkelstein and McGarry, 2006).

Many works are devoted to studying the optimal strategies and actions of market agents suffering from information asymmetry (the underinformed agents). The concept of signaling, introduced in the seminal work of M. Spence (Spence, 1973), is widely used to describe one of the major classes of filtering mechanisms on the market with unobservable characteristics. Since then, the principles of reliable signaling are developed (de Haan et al., 2011; Alós-Ferrera and Prat, 2012), and classification of equilibria in signaling problems is given (Cho and Kreps, 1987; Cobb and Basuchoudhary, 2009). In some works, a regulatory signaling analysis is carried out (Licari and Meier, 2000; Austen-Smith and Fryer, 2005; Pecorino and Van Boening, 2018). The concept and methods of screening in the conditions of asymmetric information, including exclusive and competitive screening, have been developed (Riley, 2001; Strausz, 2017; Lester et al., 2019). Signaling mechanisms are widely used in nowadays economy, particularly in labor markets, commodity markets, as well as signaling in economic policy.

Our model is a natural development of these works to the market of innovation projects. We use the Bayesian game model for the principal-agent system as a basis, and then we adopted it for the market of innovation projects by adding a system of specific constraints. This allowed us to obtain the boundary conditions for the investor's decision depending on the characteristics of both players. Formal representation of these conditions as inequalities allows us to obtain a quantitative expression for assessing the effectiveness of the final negotiation strategy (be it pure or mixed one) for the investor. It is important to note that the variables included in these inequalities are either objectively observable during the negotiation process (such as the proposed share of revenue or ownership) or directly assessed by the players (for example, the subjective probabilities of project success). It follows from this that we get rid of the information asymmetry in our model. The remaining uncertainty is inherent and irreducible.

## 3 Bayesian approach

The interactions between economic agents with different nontransferable and unobservable knowledge (knowledge asymmetry) can be simulated by a game with incomplete information (Harsanyi, 1967, 1968). In this case, the hidden knowledge determines the so-called “type” of a particular player.

Consider such a game in a form given in (Gurtuev et al., 2018). Let us say that players can have private unobservable information, either a priori or interim, and have quasi-linear preferences.

Such a game can be written as a finite game:

(1) $$G_{h}={\left(N,S_{i},T_{i},p_{i},u_{i}\right)}_{i\in N}$$

where:

*N* is the set of players (an innovator and an investor);

*S*_*i*_ – the set of strategies (participation or non-participation for an innovator, the particular investment support for an investor);

*T*_*i*_ – the set of player types;

*p*_*i*_ – players' ideas about the distribution of player types;

*u*_*i*_ – players' benefits. It is worth noting that in the case of fake innovators, the benefits for both players are less than 0;

There is a widely used natural assumption that in a Bayesian game, every player, at least *ex interim* knows both her type and the payouts of the game. However, in (1), a particular player does not know her type *a priori*. Instead, she has a probabilistic assessment, independent from the evaluations of her type by other players. Possibly for an innovator, it is plausible to assume at least the direction of its evaluation bias (towards the higher type *H*), but hardly more than that (Gurtuev et al., 2018).

Contrary to the single-turn simultaneous games, for a repetitive game with communication, the difference between players intrinsically knowing their types and having merely probabilistic assessments affects the players' strategies and the possible equilibria significantly. In short, using such games to model bargaining interaction between investors and innovators, we can describe a mechanism for investment in innovation projects as a closed system of consecutive repetitive games with knowledge asymmetry, deferred payouts, and Bayesian recalculation of player's type assessment.

## 4 The objective of the study

One of the important problems in investment in innovation projects is the problem of filtering off fraudulent projects. We consider here projects based on deliberately unrealizable concepts and aimed only at obtaining funding, and then after a few years, either report on the impossibility of carrying out the project for reasons beyond the control of the “innovator” or issue a product that does not possess the stated characteristics.

We define a fraudulent project as a project aimed only at obtaining non-repayable funding, a project that, by the estimates of its creators, does not have a profitable future.

## 5 Model and results

### 5.1 Assumptions

Let us set the initial assumptions for the base model:

1. There are two classes of players: innovators (Ei) and investors (Fi);
2. There are two types of projects: real projects (H) and fake projects (L);
3. Time t is discrete. During one period of time *t*_*i*_*,* there is one move of the “innovator-investor” game for each project. Decisions within one game move are made simultaneously;
4. All agents share common information and have their own private information;
5. There is no inflation;
6. There is no credit; all agents have an initial budget and a strict budget constraint;
7. Every non-funded project is presented to at least one investor in each period *t*_*i*_. A project can be presented to any number of investors within one period of time;
8. The presentation of the project to the investor entails expenses from the innovator (no cheap talk).
9. An innovator is optimistic (his *a priori* evaluation of her type is higher than her *a priori* evaluation of the average type of projects in any set of projects, including its own) and risk-neutral. One innovator represents only one project at a time.
10. The investor is risk-seeking (has convex expected utility function)

### 5.2 Strategies

The possible players' strategies in such a system can be represented as a set of decisions for the following variables:

For the innovator:

• requested funding amount (estimated minimum cost *C*_*min*_ and premium cost *C*_*extra*_). Let us assume that work remuneration and other direct benefits received by an innovator in the course of project implementation are included in *C*_*extra*_, not in *C*_*min*_;
• participation in costs, *C*_*e*_ (the innovator participates in project expenses or does not participate);
• proposed investor's share in the project, *S*;
• *a priori* probabilistic estimation of the project success, *q* < 1;
• estimation of the average future project value (NPV) in case of successful implementation, *v*_*e*_;
• estimation of the average deviation from the future project value in case of successful implementation, *σv*_*e*_;
• estimated time to a successful implementation of the project, *t*_*e*_*.*

For the investor:

• type of project funding (full financial support, partial financing with a particular share of *s*_*i*_, refusal of financing);
• bargaining (to bargain or not to bargain with the innovator);
• *a priori* probabilistic estimation of the project success, *p* < 1;
• estimation of the average future project value (NPV) in case of successful implementation, *v*_*i*_;
• estimation of the average deviation from the future project value in case of successful implementation, *σv*_*i*_;
• estimated time to a successful implementation of the project, *t*_*e*_;
• discount parameter for the uncertainty of the future, *r*_*t*_ < 1.

### 5.3 Player types

Let us consider two types of innovators – honest ones (H) and fraudulent ones (L). Let us assume that an honest innovator has the following characteristics:

• C_min_ > 0;
• C_extra_ > 0, 0 < C_extra_ < C_min_;
• C_e_ << C_min_;
• 0 < q < 1, 0 < p < ½;
• v_e_ >> (C_extra_ + C_min_);
• t_e_ > 0;
• σv_min_ < σv_e_ < v_e_, σv_min_ < (C_extra_ + C_min_).

σ_min_ here is considered as the risk boundary between a traditional industry investment project and an innovation project. When investing in a non-innovation project, the cost size and structure are known at the beginning and usually do not alter much, but for an innovation project, almost everything – technology, materials, staff, even target market can be significantly changed during the project development.

Let us assume that a fake innovator has the following characteristics:

• C_min_ = 0;
• C_extra_>0;
• C_e_ << C_extra_;
• q = 0;
• v_e_ = 0;
• t_e_ > 0 (in this case, this parameter does not matter. Usually the more t_e_ is, the more C_extra_ is, but C_extra_ here is already directly taken into account);
• σv_e_ = 0.

Let there be only one type of investor.

### 5.4 The normal form of the initial game model

Let the estimation of the project cost in case of successful implementation can be agreed between the investor and the innovator (as a random variable with the normal distribution, expected value equal to *v* and the standard deviation equal to *σv*). However, *a priori* estimations of the chances of project success by the investor and innovator are still different.

The estimation of the project cost consists of two parts – the necessary costs (minimal costs for the project implementation) and extra costs (allowing some premium spending, publicity, and better operating conditions). Bargaining over additional costs can be described by a modified principal-agent model.

Different players have different knowledge about the payment matrix in the model – the investor believes that the project would be successful with probability *p*, and the innovator – with probability *q.* So, for the investor, the expected payouts (both the investor's and the innovator's) will be different than for the innovator. Moreover, *a posteriori* equilibrium will be different from the *interim* equilibrium. We assume that the players know about this situation, similar to Milgrom's game with a sophisticated novice (Milgrom, 2008).

In normal form, one period of the game looks like the following (Table 1):

**Table 1:** One period of the Bayesian game between innovator and investor.

	Support (partial or full, *S* = Σ*s*_*i*_<1)	Decline	Bargain (*P*^*b*^<1, 0 < *s*^*b*^_*i*_<1, *S* = Σ*s*_*i*_ + *s*^*b*^_*i*_ <1)
Honest – Cost participation (***C***_***e***_)	*p*_∗_*s*_*i*∗_*v*_*i*_ – *s*_*i*∗_(*C*_*min*_ + *C*_*extra*_-*C*_*e*_) *q*_∗_(1-*S*)_∗_*v*_*e*_ –*C*_*e*_ + *C*_*extra*_	0 0	*P*^*b*^_∗_[*p*_∗_(*s*_*i*_ + *s*^*b*^_*i*_)_∗_*v*_*i*_ – *s*_*i*∗_(*C*_*min*_ + *C*_*extra*_-*C*_*e*_)] *P*^*b*^_∗_*q*_∗_(1-*S-s*^*b*^_*i*_)_∗_*v*_*e*_ –*C*_*e*_ + *C*_*extra*_
Honest – Costs already incurred (***C***_***e***_)	*p*_∗_*s*_*i*∗_*v*_*i*_ – *s*_*i*∗_(*C*_*min*_ + *C*_*extra*_-*C*_*e*_) *q*_∗_(1-*S*)_∗_*v*_*e*_ –*C*_*e*_ + *C*_*extra*_	0 –***C***_***e***_	*P*^*b*^_∗_[*p*_∗_(*s*_*i*_ + *s*^*b*^_*i*_)_∗_*v*_*i*_ – *s*_*i*∗_(*C*_*min*_ + *C*_*extra*_-*C*_*e*_)] *P*^*b*^_∗_*q*_∗_(1-*S-s*^*b*^_*i*_)_∗_*v*_*e*_ –*C*_*e*_ + *C*_*extra*_
Honest – No cost participation	*p*_∗_*s*_*i*∗_*v*_*i*_ – *s*_*i*∗_(*C*_*min*_ + *C*_*extra*_) *q*_∗_(1-*S*)_∗_*v*_*e*_ + *C*_*extra*_	0 0	*P*^*b*^_∗_[*p*_∗_(*s*_*i*_*+ s*^*b*^_*i*_)_∗_*v*_*i*_ – *s*_*i*∗_(*C*_*min*_ + *C*_*extra*_)] *P*^*b*^_∗_*q*_∗_(1-*S-s*^*b*^_*i*_)_∗_*v*_*e*_ + *C*_*extra*_
Fake – Cost participation	*p*_∗_*s*_*i*∗_*v*_*i*_ – *s*_*i*∗_(*C*_*min*_ + *C*_*extra*_-*C*_*e*_) –*C*_*e*_ + *C*_*extra*_	0 0	*P*^*b*^_∗_[*p*_∗_(*s*_*i*_ + *s*^*b*^_*i*_)_∗_*v*_*i*_ – *s*_*i*∗_(*C*_*min*_ + *C*_*extra*_-*C*_*e*_)] –*C*_*e*_ + *C*_*extra*_
Fake – Costs already incurred	*p*_∗_*s*_*i*∗_*v*_*i*_ – *s*_*i*∗_(*C*_*min*_ + *C*_*extra*_-*C*_*e*_) –*C*_*e*_ + *C*_*extra*_	0 –***C***_***e***_	*P*^*b*^_∗_[*p*_∗_(*s*_*i*_ + *s*^*b*^_*i*_)_∗_*v*_*i*_ – *s*_*i*∗_(*C*_*min*_ + *C*_*extra*_-*C*_*e*_)] –*C*_*e*_ + *C*_*extra*_
Fake – No cost participation	*p*_∗_*s*_*i*∗_*v*_*i*_ – *s*_*i*∗_(*C*_*min*_ + *C*_*extra*_) *C*_*extra*_	0 0	*P*^*b*^_∗_[*p*_∗_(*s*_*i*_*+ s*^*b*^_*i*_)_∗_*v*_*i*_ – *s*_*i*∗_(*C*_*min*_ + *C*_*extra*_)] *C*_*extra*_

where:

***C***_***min***_ is the evaluation of minimum costs;

***C***_***extra***_ – the evaluation of the extra budget, including remuneration and other direct benefits received by an innovator during the project implementation;

***C***_***e***_ – participation in expenses by innovator;

***S*** – the share offered to the investor in the project;

***p***, ***q*** – *a priori* evaluations of the probability of the project's success by the investor and the innovator (***p***, ***q*** <1), respectively;

***v*** – expected NPV of the project in the case of success;

***s***_***i***_ – the share of participation in the financing of the project for investor ***i***;

***P***^***b***^ – an estimate of the likelihood of success in the bargain (success here means that the innovator agrees to the investor's counter-offer);

***s***^***b***^_***i***_ – additional future payouts to investor ***i*** without increasing the project financing (the result of bargaining).

### 5.5 Final model

Let us assume that the share of fraudulent projects in the total number of projects is *p*^*f*^, 0 < *p*^*f*^ <1, and the probability of identifying a fraudulent project at the project review stage is *r*^*f*^ (with zero false positive signal probability), *r*^*f*^ <1.

Let ***C*** be the total project costs claimed by the innovator (let us assume that the investor cannot distinguish minimum budget ***C***_***min***_ from extra costs ***C***_***extra***_). This assumption looks realistic due to the asymmetry of knowledge between the innovator and the investor.

Then, from the investor's point of view, the game looks like the following:

With a probability of ***g*** = *(1-p*^*f*^*)/(1-r*^*f*^*p*^*f*^*)*, he plays with the H-type innovator (Table 2).

**Table 2:** The game between the investor and H-type (“honest”) innovator.

	Support (partial or full, *S* = Σ*s*_*i*_<1)	Decline	Bargain (*P*^*b*^<1, 0 < *s*^*b*^_*i*_<1, *S*^*b*^ = Σ*s*^*b*^_*i*_ <1, *S* + *S*^*b*^ <1)
Cost participation (***C***_***e***_)	*p* ∗ *s*_*i*_ ∗*v* ∗*f(σv)* – (*s*_*i*_/*S*)∗(*C*–*C*_*e*_) *q*_∗_(1-*S*)_∗_*v* – *C*_*e*_	0 0	*P*^*b*^_∗_[*p*_∗_(*s*_*i*_ + *s*^*b*^_*i*_)_∗_*v ∗f(σv)* –(*s*_*i*_/*S*)∗(*C*–*C*_*e*_)] *P*^*b*^∗[*q*_∗_(1-*S-S*^*b*^)_∗_*v* –*C*_*e*_]
Costs already incurred (***C***_***e***_)	*p* ∗ *s*_*i*_ ∗*v* ∗*f(σv)* – (*s*_*i*_/*S*)∗(*C*–*C*_*e*_) *q*_∗_(1-*S*)_∗_*v* – *C*_*e*_	0 –***C***_***e***_	*P*^*b*^_∗_[*p*_∗_(*s*_*i*_ + *s*^*b*^_*i*_)_∗_*v ∗f(σv)* –(*s*_*i*_/*S*)∗(*C*–*C*_*e*_)] *P*^*b*^∗[*q*_∗_(1-*S-S*^*b*^)_∗_*v*] –*C*_*e*_
No cost participation	*p* ∗ *s*_*i*_ ∗*v* ∗*f(σv)* – (*s*_*i*_/*S*)∗*C* *q*_∗_(1-*S*)_∗_*v*	0 0	*P*^*b*^_∗_[*p*_∗_(*s*_*i*_ + *s*^*b*^_*i*_)_∗_*v ∗f(σv)* –(*s*_*i*_/*S*)∗*C*] *P*^*b*^∗[*q*_∗_(1-*S-S*^*b*^)_∗_*v*]

With probability of ***1-g*** = *[(1-r*^*f*^*) p*^*f*^*]/(1-r*^*f*^*p*^*f*^*)*, the investor plays with the fake innovator (Table 3):

**Table 3:** The game between the investor and L-type (“fake”) innovator.

	Support (partial or full, *S* = Σ*s*_*i*_<1)	Decline	Bargain (*P*^*b*^<1, 0 < *s*^*b*^_*i*_<1, *S*^*b*^ = Σ*s*^*b*^_*i*_ <1, *S* + *S*^*b*^ <1)
Cost participation (***C***_***e***_)	– (*s*_*i*_/*S*)∗(*C*–*C*_*e*_) *C*–*C*_*e*_	0 0	*P*^*b*^*∗*[–(*s*_*i*_/*S*)∗(*C*–*C*_*e*_)] *P*^*b*^∗[*C*–*C*_*e*_]
Costs already incurred (***C***_***e***_)	– (*s*_*i*_/*S*)∗(*C*–*C*_*e*_) *C*–*C*_*e*_	0 –***C***_***e***_	*P*^*b*^*∗*[ –(*s*_*i*_/*S*)∗(*C*–*C*_*e*_)] *P*^*b*^∗*C*–*C*_*e*_
No cost participation	– (*s*_*i*_/*S*)∗*C* *C*	0 0	*P*^*b*^*∗*[ –(*s*_*i*_/*S*)∗*C*] *P*^*b*^∗*C*

Let us consider now the investor's strategy.

#### 5.5.1 The case of an H-type (“honest”) innovator

Let us denote the investor's payout as ***B***_***i***_ **=** *p∙s*_*i*_*∙v∙f(σv)*, investor's expenses as ***С***_***i***_ *= (s*_*i*_*/S)∙(C–C*_*e*_*)*, and the innovator's payout as ***B***_***e***_ = *q∙(1-S)∙v*, we get an estimation of payouts in one period of the investor-innovator game (Table 4).

**Table 4:** Estimation of payouts in one period of the investor-innovator game.

	Support (partial or full, *S* = Σ*s*_*i*_<1)	Decline	Bargain (*P*^*b*^<1, 0 < *s*^*b*^_*i*_<1, *S*^*b*^ = Σ*s*^*b*^_*i*_ <1, *S* + *S*^*b*^ <1)
Cost participation (***C***_***e***_)	$B_{i}-C_{i}$ $B_{e}-C_{e}$	0 0	$B_{i}-C_{i}-\left[\left(1-P^{b}\frac{s_{i}+s_{i}^{b}}{s_{i}}\right)B_{i}-\left(1-P^{b}\right)C_{i}\right]$ $B_{e}-C_{e}-\left[\left(1-P^{b}\frac{1-S-S^{b}}{S}\right)B_{e}-\left(1-P^{b}\right)C_{e}\right]$
Costs already incurred (***C***_***e***_)	$B_{i}-C_{i}$ $B_{e}-C_{e}$	0 –*C*_*e*_	$B_{i}-C_{i}-\left[\left(1-P^{b}\frac{s_{i}+s_{i}^{b}}{s_{i}}\right)B_{i}-\left(1-P^{b}\right)C_{i}\right]$ $B_{e}-C_{e}-\left(1-P^{b}\frac{1-S-S^{b}}{S}\right)B_{e}$
No cost participation	*B*_*i*_ –(*C*_*i*_ +(*s*_*i*_/*S*)∗*C*_*e*_) $B_{e}$	0 0	$B_{i}-\left(C_{i}+\frac{s_{i}}{S}C_{e}\right)-\left[\left(1-P^{b}\frac{s_{i}+s_{i}^{b}}{s_{i}}\right)B_{i}-\left(1-P^{b}\right)\left(C_{i}+\frac{s_{i}}{S}C_{e}\right)\right]$ $B_{e}-\left(1-P^{b}\frac{1-S-S^{b}}{S}\right)B_{e}$

Thus, we can write conditions for the bargain strategy preference for the investor here as follows:

**Table:** 

Cost participation or cost incurred (*C*_*e*_)	$\left(1-P^{b}\frac{s_{i}+s_{i}^{b}}{s_{i}}\right)\leq \frac{\left(1-P^{b}\right)C_{i}}{B_{i}}$
No cost participation	$\left(1-P^{b}\frac{s_{i}+s_{i}^{b}}{s_{i}}\right)\leq \frac{\left(1-P^{b}\right)\left(C_{i}+\frac{s_{i}}{S}C_{e}\right)}{B_{i}}$

For the innovator, we can write conditions for the bargain strategy preference in the game as follows:

**Table:** 

Cost participation (*C*_*e*_)	$P^{b}\frac{1-S-S^{b}}{S}\geq 1+\frac{\left(1-P^{b}\right)C_{e}}{B_{e}}$
Costs already incurred (***C***_***e***_)	$P^{b}\frac{1-S-S^{b}}{S}\geq 1$
No cost participation	$P^{b}\frac{1-S-S^{b}}{S}\geq 1$

Since $\frac{\left(1-P^{b}\right)C_{e}}{B_{e}}>0$, the innovator's participation in project expenses increases the area of the income distribution, in which the bargaining strategy is preferable for the H-type innovator.

#### 5.5.2 The case of an L-type (“fake”) innovator

In the case of a fake innovator, we can assume that she always agrees to bargain (*P*_*b*_ = 1), and thus simplify the game to the following:

**Table:** 

	Support (partial or full, *S* = Σ*s*_*i*_<1)	Decline
Cost participation (***C***_***e***_)	– (*s*_*i*_/*S*)∗(*C*–*C*_*e*_) *C*–*C*_*e*_	0 0
Costs already incurred (***C***_***e***_)	– (*s*_*i*_/*S*)∗(*C*–*C*_*e*_) *C*–*C*_*e*_	0 –*C*_*e*_
No cost participation	– (*s*_*i*_/*S*)∗*C* *C*	0 0

Here we have two equilibria – (*Cost participation*, *Decline*) and (*No cost participation*, *Decline*), since *Decline* strategy is dominant for the investor, and strategy *Costs already incurred* for the fake innovator is dominated by both other strategies.

Between game periods, ***p*** and ***q*** are recalculated by investor and innovator, correspondingly. This recalculation follows the *regret matching* rule (Porter et al., 2008), i.e., the probability that the agent ***i*** will have type ***s*** in the period t + 1 by its private expectation is equal to:

(2) $$\sigma_{i}^{t+1}\left(s\right)=\frac{R^{t}\left(s\right)}{\sum_{s^{\prime }\in S_{i}}R^{t}\left(s^{\prime }\right)}$$

where:

s′ – all other agent types, different from ***s***,

R^t^(s) = max (α^t^(s) – α^t^, 0) – loss of payout from the fact that the agent did not evaluate his type as ***s***.

## 6 Conclusion and discussion

We propose a new game-theoretic model for simulating the interaction of the investor and the innovator, with subjective probabilities recalculated using the regret matching rule. In our model, the uncertainties for both players are reduced to the subjective probabilities of future events. This, in contrast to the “principal-agent” models, makes it possible to exclude strategies with explicit deceptive manipulation of information. At the same time, like in hypergame models (Cho et al., 2019), we place the possibility of being deceived outside the game into the probabilistic assessment ***g*** and assume that within one period of the game it is impossible to reduce the epistemic uncertainty associated with it.

For the presented model, we obtain the conditions for the optimal strategies of all types of players for the current game period. The adjustment of probabilistic estimates during the game allows players to match an equilibrium solution in the course of bargaining.

The conditions inequalities, which formally represent the explicit optimal strategies of the players, allow the counterparties to understand the ways to reach an agreement in the course of real negotiations better.

The essential feature of the innovation process is the uniqueness of each project. The future state of the market for such projects is affected by too many uncertain factors, either of technological or social origin, so the common methods cannot obtain a robust project evaluation. An important consequence of this is the difficulty of filtering the fake innovators out.

Another issue worth noting is that the specificity of the investment in innovation projects lies in the fact that some mechanism is needed to distinguish projects that have low *a priori* probability of type H (pessimistic innovators) from projects that are obviously of type L (fake innovators). “Principal-agent” models assume that agents know their type at least *ex interim*, and the menu of contracts is based on the unprofitability to the agent of the low type to signal the high type. For investment in innovation projects, such a menu will cut off not only fake innovators but also pessimistic innovators.

The model of interaction between investor and innovator presented here allows quantifying the strategic parameters for both players to find equilibrium solutions. More importantly, for the real problems of financing innovation projects, it allows finding rational modes for screening fake innovators based on repetitive adjustment of players' probabilistic estimations during bargaining.

However, the presented mechanism has some limitations in practical usage. In this part of the work, we want to highlight and distinguish two potential sources of such limitations that should be addressed independently and differently during the development of a field solution for a particular innovator-investor bargain.

The first source is the optimistic bias that is inherent to innovators. It leads not only to overvaluing the expectation of project's success probability ***q***, but also to underestimation of future minimum costs ***C***_***min***_. This can lead to overinvestment and should be addressed via particular institutional means, based on a further special study. The second one is that the minimum project budget can be very high, and the condition of cost participation became unrealistic. Although the proposed model is still reliable in its base shape, the given solution is not suitable for such a situation.

As for future implications of the presented model and approach, we could state that it seems possible to build an effective institutional mechanism for coordinating the interests of the innovator and investor with the filter of pseudo-innovators. Such a mechanism can be a direct incentive-compatible mechanism (Gurtuev, 2018). However, a thorough study of limitations and possible shapes of a particular realization of a direct incentive-compatible mechanism with the filter of fake innovators is yet to be carried out.

## Declarations

### Author contribution statement

Alim Gurtuev: Conceived and designed the experiments; Analyzed and interpreted the data; Contributed reagents, materials, analysis tools or data; Wrote the paper.

Elena Derkach: Contributed reagents, materials, analysis tools or data.

Salima Makhosheva, Zaur Ivanov: Analyzed and interpreted the data; Contributed reagents, materials, analysis tools or data.

### Funding statement

This work was supported by 10.13039/501100002261RFBR, research projects No. 19-010-00376 A, 19-010-00577 A, and 20-010-00269 A.

### Data availability statement

No data was used for the research described in the article.

### Declaration of interests statement

The authors declare no conflict of interest.

### Additional information

No additional information is available for this paper.
